# Supplementary material for: Disentangling phonology from phonological short-term memory in Alzheimer’s disease phenotypes
Source: Alzheimers Res Ther. 2025 Sep 30;17:206. doi: 10.1186/s13195-025-01856-x (PMC12482198; doi:10.1186/s13195-025-01856-x)
Supplement: Supplementary file 1 — Supplementary Material 1 [file 13195_2025_1856_MOESM1_ESM.pdf]

Supplementary Table 1 Detailed phonological error patterns in patients during PALPA non-word repetition

Participant ID	1	2	3	4	5	6	7	8	9	10	11	12	13	14	15	16	17	18	19	20	21	22	23	24	25	26	27
Diagnosis	Lv+	lv	tAD	tAD	Lv+	lv	tAD	Lv	Lv+	lv	Lv	tAD	Lv+	Lv+	tAD	tAD	Lv+	lv	tAD	Lv+	Lv	lv	lv	tAD	Lv	Lv+	tAD
Hearing loss	Yes	No	Yes	Yes	Yes	Yes	Yes	No	No	No	Yes	Yes	No	No	No	No	No	No	No	No	No	No	No	No	No	No	No
Non-word repetition total	7	11	14	17	19	19	19	20	21	21	21	22	22	23	23	24	25	25	25	26	27	27	27	27	27	29	29
**Types of errors**																											
Substitutions (subs)	16	12	9	11	5	10	9	7	4	7	8	7	7	7	7	5	4	5	5	2	2	3	3	3	3	1	1
Omissions	2	1	3	2	3		1	2	2	1		1	1			1	1										
Additions	2	4	2		1			1		1	1										1						
Combinations	2	2	2		2	1			3											2							
O+S	1				2	1			2																		
A+S	1	1	1						1											2							
A+T		1																									
T+S			1																								
Lexicalisation (%)	11 (48%)	0 (0%)	3 (19%)	6 (46%)	2 (18%)	2 (18%)	3 (23%)	0 (0%)	4 (44%)	3 (33%)	1 (13%)	2 (25%)	2 (25%)	4 (57%)	2 (28%)	3 (50%)	2 (20%)	1 (20%)	2 (40%)	2 (50%)	3 (100%)	2 (66%)	3 (100%)	1 (33%)	0 (0%)	0 (0%)	1 (100%)
**Detailed error analysis**																											
**Position**																											
Initial	10	2	6	7	3	1	4	3	4	2	1	3		3	4	1	1		3		1	2		1		1	
Medial	7	9	4	3	6	7	3	4	3	3	3	5	4	2	2	2	2	3	1	4	2	1	2	2	1		
Final	2	3	2	1	2	2	2	3	2	3	1		2	2			1								1		
Unclassifiable	4	5	4	2		1	2			1	4		2		1	3	1	2	1				1		1		1
**Phoneme(s)**																											
Single cons	4	8	5	5	7	8	7	5	6	7	5	5	2	5	4	2	2	3		1	1	1	1	1	1	1	
Single vowel	3	4		2			2					2	2	1	1		1		2	1	1	2	1	2	1		
Cons clusters	6		4	4	4			2	1	1		1	1	1	1	1	1		1								
Multiple phonemes	10	7	7	2		3	2	3	2	1	9		3		1	3	1	2	2	2	1		1		1		1
**Subs**																											
Stopping		1		1	1	2	1	1	1	1	1																
Fronting		1		1	2			1	1	1	1	1		2				1		1	1		1				
Backing		1	1	1		1	1	1		1		1			1												
Deaffrication																											
Affrication		1		1		2	1	1		1							1								1		
Alveolar	1	1								1	1		1	2		1											
Depalatal																											
Labialisation					1	1	1	1					1														
Gliding		1																									
Voicing		1									1																
Vowel sub*	3	4		2			2					2	2	1	1		1		2	1		2	1	2	1		

Coalescence	3		1	1															1		1						
Denasalisation				1												1											
Assimilation																		1									
Others	9	1	7	3	2	4	3	2	2	2	4	3	3	2	5	3	2	3	2			1	1	1	1	1	1
**Omissions**																											
Final cons		1	1		1		1	1	1	1			1				1										
Initial cons	1		1	1					1							1											
Cluster reduction	1		1	1	2			1				1															

The Supplementary Table above summarises the phonological errors made by each patient participant during the non-word repetition task. The anonymised participant IDs were rank ordered from most (Participant #1) to least impaired (Participant #27) on this task. There was considerable variability across participants, and the degree of impairment was not specific to a diagnostic group with the diagnoses interspersed across the rank orders. All participants made at least one phonological error with a range of one (Participants #26 and #27) to 23 (Participant #1) errors across the entire sample. Substitutions accounted for most errors (76% of all errors) and did not appear to be specific to a type of phonological error such as stopping and backing. Omissions, which accounted for 10% of all errors, were limited to cluster reduction, initial and final consonant deletions. Additions accounted for 6% of all errors and transposition errors were not observed. Lexicalisation errors which occurred in 31% of all errors was found to be variable. For example, Participant #1, who made the most phonological errors, lexicalised 48% of the errors whereas the next patient who made the second most errors across the whole group (Participant #2) did not lexicalise any of their errors. Supplementary Table 2 shows the type and details of the errors made by controls during the non-word repetition task. Substitutions accounted for most errors (87%) and about 35% of these were lexicalisation errors. Note: Vowel substitutions refer to errors involving the production of a non-target vowel (e.g., /ae/ for /ɛ/) and should be interpreted differently from another phonological error pattern known as ‘vowelisation’ which involves the substitution of a vowel for a liquid sound. Alveolar, alveolarisation; A+S, combination of addition and substitution; A+T, combination of addition and transposition; cons, consonant; depalatal, depalatalisation; lv+, lvPPA+; O+S, combination of omission and substitution; tAD, typical amnestic Alzheimer’s disease; T+S, combination of transposition and substitution.

Supplementary Table 2 Detailed phonological error patterns in healthy controls during PALPA non-word repetition

Control participant ID	1	2	3	4	5	6	7	8	9	10	11	12
Hearing loss	Yes	No	Yes	Yes	Yes	No	No	No	No	No	No	No
Non-word repetition total	17	19	24	25	25	25	27	27	28	28	28	30
**Type of errors**												
Substitutions	12	8	5	5	5	5	2	3	1	2	2	
Omissions		3										
Additions	1								1			
Transpositions							1					
Combinations:<br>O+S			1									
Lexicalisation (%)	3 (25%)	4 (36%)	2 (40%)	1 (20%)	1 (20%)	1 (20%)	1 (33%)	1 (33%)	2 (100%)	2 (100%)	2 (100%)	
**Detailed error analysis**												
**Position:**												
Initial	4	5		3					1	1		
Medial	6	4	4	1	2	3	2	2		1	2	
Final	1	2	1	1	1			1	1			
Unclassifiable	2		1		2	2	1					
**Phoneme(s):**												
Single consonant	7	5	3	2	3	3	2	2	1	1	1	
Single vowel	2	2		3				1		1	1	
Consonant clusters	1	3	1						1			
Multiple phonemes	3	1	2		2	2	1					
**Substitutions:**												
Stopping	1	1		1	1			1	1			
Fronting	1	1	1			1	1	1		1	1	
Backing	1	2										
Deaffrication												
Affrication				1								
Alveolarisation					1							
Depalatalisation												
Labialization	1											
Gliding												
Voicing			1			1						
Vowel sub*	3	2	1	3		1		1		1	1	
Coalescence												
Denasalisation												
Assimilation												
Others	5	2	2		3	2	1					
**Omissions:**												
Final consonant		1										
Initial consonant		2										
Cluster reduction												

Supplementary Table 3 Bayesian correlations between non-word and multisyllabic word/phrase repetition and connected speech derived measures

Connected speech derived measure	All groups		tAD		lvPPA		lvPPA+	
	PALPA non-word repetition	ACE-R multisyllabic word/phrase repetition	PALPA non-word repetition	ACE-R multisyllabic word/phrase repetition	PALPA non-word repetition	ACE-R multisyllabic word/phrase repetition	PALPA non-word repetition	ACE-R multisyllabic word/phrase repetition
Phonological paraphasias per 100 words	*r* = -0.24, *P* = 0.23, BF = 20.48	*r* = -0.43, *P* = 0.03, BF = 2.49	*r* = 0.21, *P* = 0.60, BF = 0.46	*r* = 0.23, *P* = 0.55, BF = 0.48	*r* = -0.25, *P* = 0.48, BF = 0.48	*r* = -0.42, *P* = 0.23, BF = 0.73	***r* = -0.80, *P* = 0.02, BF = 4.64**	*r* = -0.24, *P* = 0.60, BF = 0.52
Fillers per 100 words	*r* = 0.29, *P* = 0.14, BF = 0.66	*r* = -0.27, *P* = 0.18, BF = 0.58	*r* = 0.64, *P* = 0.07, BF = 1.81	*r* = 0.47, *P* = 0.21, BF = 0.82	*r* = 0.30, *P* = 0.39, BF = 0.54	*r* = -0.41, *P* = 0.24, BF = 0.72	*r* = 0.22, *P* = 0.61, BF = 0.48	***r* = 0.90, *P* = 0.006, BF = 10.07**
Repaired sequences per 100 words	*r* = -0.08, *P* = 0.71, BF = 0.27	*r* = -0.45, *P* = 0.02, BF = 2.97	*r* = 0.14, *P* = 0.72, BF = 0.43	*r* = 0.06, *P* = 0.88, BF = 0.41	*r* = 0.05, *P* = 0.90, BF = 0.39	*r* = -0.44, *P* = 0.20, BF = 0.80	*r* = -0.74, *P* = 0.04, BF = 2.74	*r* = 0.53, *P* = 0.23, BF = 0.87
Pauses per 100 words	*r* = -0.20, *P* = 0.33, BF = 0.38	***r* = -0.46, *P* = 0.02, BF = 3.28**	*r* = -0.47, *P* = 0.20, BF = 0.84	*r* = 0.09, *P* = 0.81, BF = 0.42	*r* = -0.16, *P* = 0.65, BF = 0.42	*r* = -0.53, *P* = 0.12, BF = 1.17	*r* = -0.36, *P* = 0.39, BF = 0.60	*r* = 0.42, *P* = 0.35, BF = 0.67
Speech to pause ratio	*r* = 0.35, P = 0.07, BF = 1.09	*r* = 0.09, *P* = 0.65, BF = 0.27	*r* = 0.46, *P* = 0.21, BF = 0.81	*r* = -0.04, *P* = 0.92, BF = 0.41	*r* = 0.34, *P* = 0.33, BF = 0.59	*r* = 0.21, *P* = 0.57, BF = 0.45	*r* = 0.37, *P* = 0.36, BF = 0.62	*r* = -0.34, *P* = 0.46, BF = 0.58
Words to non-word utterances ratio	*r* = 0.004, *P* = 0.99, BF = 0.24	*r* = 0.29, *P* = 0.15, BF = 0.64	*r* = -0.70, *P* = 0.04, BF = 2.59	*r* = -0.30, *P* = 0.43, BF = 0.53	*r* = 0.02, *P* = 0.97, BF = 0.39	***r* = 0.73, *P* = 0.02, BF = 4.66**	*r* = 0.60, *P* = 0.12, BF = 1.24	*r* = -0.57, *P* = 0.19, BF = 0.97
Words per minute	*r* = 0.09, *P* = 0.65, BF = 0.26	*r* = 0.31, *P* = 0.12, BF = 0.75	*r* = 0.38, *P* = 0.31, BF = 0.64	*r* = 0.34, *P* = 0.37, BF = 0.58	*r* = -0.29, *P* = 0.42, BF = 0.52	*r* = 0.17, *P* = 0.63, BF = 0.43	*r* = -0.38, *P* = 0.36, BF = 0.62	*r* = -0.49, *P* = 0.27, BF = 0.78
Total utterances per minute	*r* = -0.01, *P* = 0.97, BF = 0.24	*r* = -0.06, *P* = 0.77, BF = 0.25	***r* = 0.73, *P* = 0.03, BF = 3.43**	*r* = 0.23, *P* = 0.56, BF = 0.47	*r* = -0.37, *P* = 0.30, BF = 0.63	*r* = -0.25, *P* = 0.49, BF = 0.48	*r* = -0.07, *P* = 0.87, BF = 0.43	*r* = -0.32, *P* = 0.49, BF = 0.56
Fillers per minute	*r* = 0.37, *P* = 0.06, BF = 1.26	*r* = -0.03, *P* = 0.90, BF = 0.25	*r* = 0.59, *P* = 0.10, BF = 1.35	*r* = 0.47, *P* = 0.21, BF = 0.83	*r* = 0.28, *P* = 0.43, BF = 0.51	*r* = -0.28, *P* = 0.43, BF = 0.51	*r* = 0.37, *P* = 0.37, BF = 0.61	*r* = 0.68, *P* = 0.09, BF = 1.54
Pauses per minute	***r* = -0.44, *P* = 0.02, BF = 3.10**	*r* = -0.29, *P* = 0.15, BF = 0.66	*r* = -0.31, *P* = 0.42, BF = 0.54	*r* = 0.42, *P* = 0.26, BF = 0.71	*r* = -0.66, *P* = 0.04, BF = 2.54	*r* = -0.47, *P* = 0.17, BF = 0.90	*r* = -0.44, *P* = 0.28, BF = 0.72	*r* = 0.25, *P* = 0.59, BF = 0.52
Long (>2 seconds) pauses per minute	*r* = -0.216 *P* = 0.20, BF = 0.53	*r* = -0.18, *P* = 0.38, BF = 0.35	*r* = -0.49, *P* = 0.18, BF = 0.88	*r* = -0.02, *P* = 0.95, BF = 0.41	*r* = -0.39, *P* = 0.27, BF = 0.66	*r* = -0.08, *P* = 0.83, BF = 0.39	*r* = -0.03, *P* = 0.95, BF = 0.43	*r* = 0.05, *P* = 0.92, BF = 0.46
Short pauses (<2 seconds) per minute	*r* = -0.34, *P* = 0.08, BF = 1.03	*r* = -0.22, *P* = 0.27, BF = 0.43	*r* = 0.07, *P* = 0.85, BF = 0.41	*r* = 0.63, *P* = 0.07, BF = 1.76	*r* = -0.42, *P* = 0.22, BF = 0.75	*r* = -0.46, *P* = 0.18, BF = 0.87	*r* = -0.65, *P* = 0.08, BF = 1.63	*r* = 0.42, *P* = 0.36, BF = 0.67
Repaired sequences per minute	*r* = -0.13, *P* = 0.51, BF = 0.29	*r* = -0.35, *P* = 0.08, BF = 1.02	*r* = 0.24, *P* = 0.53, BF = 0.48	*r* = 0.15, *P* = 0.69, BF = 0.44	*r* = 0.04, *P* = 0.91, BF = 0.39	*r* = -0.38, *P* = 0.28, BF = 0.65	*r* = -0.64, *P* = 0.09, BF = 1.55	*r* = 0.24, *P* = 0.60, BF = 0.52

Note: Bayes factor greater than 3 and their associated Pearson's correlation values are bolded. ACE-R, Addenbrooke's Cognitive Examination – Revised; lvPPA, logopenic variant of primary progressive aphasia; PALPA, the Psycholinguistic Assessment of Language Processing in Aphasia; tAD, typical Alzheimer's disease.

Supplementary Table 4 Bayesian point and interval estimates of effect sizes for each longitudinal case relative to their respective diagnostic group sample

Participant (diagnostic group)	Measure (maximum score, if applicable)	Group mean (SD)	Group sample size	Initial assessment score/ value	Bayesian two-tailed probability	Bayesian point estimate of control falling below case's score (95% credible interval)	Bayesian effect size (95% credible interval)	Follow-up assessment score or value	Bayesian two-tailed probability	Bayesian point estimate (95% credible interval)	Bayesian effect size (95% credible interval)
**Cognitive assessment**											
5 (lvPPA+)	PALPA word repetition (24)	23.28 (1.85)	18	22	0.51	25.50 (11.52 to 43.39)	-0.69 (-1.20 to -0.17)	8	**< 0.001**	0.00	-8.26 (-11.04 to -5.47)
18 (lvPPA)				24	0.71	64.53 (46.17 to 80.61)	0.39 (-0.10 to 0.86)	24	0.71	64.53 (46.17 to 80.61)	0.39 (-0.10 to 0.86)
22 (lvPPA)				24	0.71	64.53 (46.17 to 80.61)	0.39 (-0.10 to 0.86)	24	0.71	64.53 (46.17 to 80.61)	0.39 (-0.10 to 0.86)
19 (tAD)		23.33 (1.25)	9	23	0.81	40.44 (17.85 to 65.91)	-0.26 (-0.92 to 0.41)	24	0.62	68.76 (42.79 to 88.94)	0.54 (-0.18 to 1.22)
24 (tAD)				24	0.62	68.76 (42.79 to 88.94)	0.54 (-0.18 to 1.22)	20	**0.04**	1.78 (0.002 to 11.24)	-2.66 (-4.08 to -1.21)
27 (tAD)				24	0.62	68.76 (42.79 to 88.94)	0.54 (-0.18 to 1.22)	24	0.62	68.76 (42.79 to 88.94)	0.54 (-0.18 to 1.22)
5 (lvPPA+)	PALPA non-word repetition (30)	22.11 (5.55)	18	19	0.59	29.63 (14.68 to 47.84)	-0.56 (-1.05 to -0.05)	5	**0.008**	0.40 (0.00 to 2.57)	-3.08 (-4.20 to -1.95)
18 (lvPPA)				25	0.62	69.06 (50.79 to 84.29)	0.52 (0.02 to 1.01)	18	0.48	24.05 (10.47 to 41.78)	-0.74 (1.26 to -0.21)
22 (lvPPA)				27	0.40	79.85 (62.70 to 92.19)	0.88 (0.32 to 1.42)	18	0.48	24.05 (10.47 to 41.78)	-0.74 (1.26 to -0.21)
19 (tAD)		22.33 (1.25)	9	25	0.08	96.13 (81.55 to 99.96)	2.14 (0.90 to 3.33)	24	0.24	87.96 (65.45 to 98.72)	1.34 (0.40 to 2.23)
24 (tAD)				27	**0.008**	99.62 (96.52 to 100)	3.74 (1.82 to 5.63)	21	0.34	17.13 (3.06 to 41.52)	-1.06 (-1.87 to -0.21)
27 (tAD)				29	**< 0.001**	99.95 (99.64 to 100)	5.33 (2.69 to 7.97)	29	**< 0.001**	99.95 (99.64 to 100)	5.33 (2.69 to 7.97)
18 (lvPPA)	ACE-R multisyllabic word/phrase repetition (6)	2.24 (1.99)	17	2	0.91	45.42 (27.58 to 64.00)	-0.12 (-0.60 to 0.36)	1	0.55	26.67 (12.81 to 46.30)	-0.62 (-1.14 to -0.09)
22 (lvPPA)				3	0.72	64.24 (45.35 to 80.77)	0.38 (-0.12 to 0.87)	0	0.29	14.51 (4.21 to 30.79)	-1.13 (-1.73 to -0.50)
19 (tAD)		5.55 (0.68)	9	6	0.55	72.62 (46.71 to 91.49)	0.66 (-0.08 to 1.37)	5	0.47	23.26 (6.06 to 48.80)	-0.81 (-1.55 to -0.03)
24 (tAD)				5	0.47	23.26 (6.06 to 48.80)	-0.81 (-1.55 to -0.03)	5	0.47	23.26 (6.06 to 48.80)	-0.81 (-1.55 to -0.03)
27 (tAD)				6	0.55	72.62 (46.71 to 91.49)	0.66 (-0.08 to 1.37)	6	0.55	72.62 (46.71 to 91.49)	0.66 (-0.08 to 1.37)
5 (lvPPA+)	Digit span forward (12)	4.72 (2.60)	18	3	0.53	26.42 (12.21 to 44.40)	-0.66 (-1.17 to -0.14)	0	0.10	4.76 (0.51 to 14.84)	-1.82 (-2.57 to -1.04)
18 (lvPPA)				5	0.92	54.12 (36.05 to 71.57)	0.11 (-0.36 to 0.57)	1	0.18	9.09 (1.86 to 22.49)	-1.43 (-2.08 to -0.76)
22 (lvPPA)				5	0.92	54.12 (36.05 to 71.57)	0.11 (-0.36 to 0.57)	4	0.80	39.55 (22.86 to 57.85)	-0.28 (-0.74 to 0.20)
19 (tAD)		8.67 (1.89)	9	10	0.52	73.84 (48.00 to 92.24)	0.70 (-0.05 to 1.42)	7	0.43	21.32 (5.03 to 46.61)	-0.88 (-1.64 to -0.09)
24 (tAD)				7	0.43	21.32 (5.03 to 46.61)	-0.88 (-1.64 to -0.09)	5	0.10	5.14 (0.11 to 21.64)	-1.94 (-3.06 to -0.78)
27 (tAD)				12	0.13	93.34 (74.85 to 99.75)	1.76 (0.67 to 2.81)	11	0.28	86.21 (62.87 to 98.19)	1.23 (0.33 to 2.09)
5 (lvPPA+)	Digit span backward (12)	2.94 (1.68)	18	1	0.28	13.84 (4.05 to 29.37)	-1.16 (-1.75 to -0.54)	0	0.11	5.34 (0.65 to 15.98)	-1.75 (-2.48 to -1.00)
18 (lvPPA)				5	0.25	87.55 (72.53 to 96.65)	1.23 (0.60 to 1.83)	2	0.59	29.66 (14.70 to 47.86)	-0.56 (-1.05 to -0.05)
22 (lvPPA)				3	0.97	51.37 (33.48 to 69.07)	0.04 (-0.43 to 0.50)	2	0.59	29.66 (14.70 to 47.86)	-0.56 (-1.05 to -0.05)
19 (tAD)		4.44 (1.71)	9	4	0.81	40.67 (18.04 to 66.12)	-0.26 (-0.91 to 0.42)	2	0.21	10.65 (0.93 to 32.30)	-1.43 (-2.35 to -0.46)
24 (tAD)				5	0.76	61.80 (36.16 to 83.90)	0.33 (-0.35 to 0.99)	4	0.81	40.67 (18.04 to 66.12)	-0.26 (-0.91 to 0.42)
27 (tAD)				7	0.19	90.33 (69.23 to 99.28)	1.50 (0.50 to 2.45)	11	**0.007**	99.67 (96.93 to 100)	3.84 (1.87 to 5.78)
1 (lvPPA+)	BNT (30)	8.50 (6.83)	18	10	0.83	58.34 (40.09 to 75.32)	0.22 (-0.25 to 0.69)	4	0.53	26.50 (12.27 to 44.49)	-0.66 (-1.16 to -0.14)
5 (lvPPA+)				1	0.30	15.01 (4.68 to 30.93)	-1.10 (-1.68 to -0.50)	0	0.24	12.12 (3.18 to 27.01)	-1.25 (-1.85 to -0.61)
18 (lvPPA)				9	0.94	52.80 (34.82 to 70.38)	0.07 (-0.39 to 0.54)	4	0.53	26.50 (12.27 to 44.49)	-0.66 (-1.16 to -0.14)
19 (tAD)		22.67 (2.54)	9	18	0.12	5.97 (0.18 to 23.55)	-1.84 (-2.92 to -0.72)	6	**< 0.001**	0.01 (0.00 to 0.04)	-6.56 (-9.77 to -3.35)
24 (tAD)				26	0.25	87.55 (64.83 to 98.60)	1.31 (0.38 to 2.20)	18	0.12	5.97 (0.18 to 23.55)	-1.84 (-2.92 to -0.72)
27 (tAD)				26	0.25	87.55 (64.83 to 98.60)	1.31 (0.38 to 2.20)	24	0.63	68.37 (42.40 to 88.67)	0.52 (-0.19 to 1.21)

1 (lvPPA+)	CSB naming (64)	37.83 (18.37)	18	47	0.63	68.34 (50.05 to 83.72)	0.50 (0.001 to 0.98)	25	0.51	25.30 (11.37 to 43.17)	-0.70 (-1.21 to -0.17)
5 (lvPPA+)				10	0.16	7.83 (1.43 to 20.63)	-1.52 (-2.19 to -0.82)	0	0.06	3.06 (0.20 to 11.08)	-2.06 (-2.88 to -1.22)
18 (lvPPA)				48	0.60	70.15 (51.93 to 85.16)	0.55 (0.05 to 1.04)	22	0.41	20.67 (8.14 to 37.90)	-0.86 (-1.40 to -0.31)
22 (lvPPA)				54	0.43	79.83 (62.67 to 92.17)	0.88 (0.32 to 1.42)	34	0.84	42.09 (25.07 to 60.33)	-0.21 (-0.67 to 0.26)
19 (tAD)		61.89 (2.18)	9	57	0.07	3.30 (0.02 to 16.69)	-2.24 (-3.49 to -0.97)	26	**< 0.001**	0.00 (0.00 to 0.00)	-16.46 (-24.40 to -8.56)
24 (tAD)				64	0.39	80.73 (55.74 to 96.00)	0.97 (0.14 to 1.75)	64	0.39	80.73 (55.74 to 96.00)	0.97 (0.14 to 1.75)
27 (tAD)				64	0.39	80.73 (55.74 to 96.00)	0.97 (0.14 to 1.75)	64	0.39	80.73 (55.74 to 96.00)	0.97 (0.14 to 1.75)
5 (lvPPA+)	Camel and cactus test (64)	48.41 (8.79)	17	56	0.41	79.58 (61.84 to 92.28)	0.87 (0.30 to 1.42)	27	**0.03**	1.46 (0.03 to 6.89)	-2.46 (-3.42 to -1.48)
18 (lvPPA)				57	0.35	82.45 (65.29 to 94.07)	0.99 (0.39 to 1.56)	46	0.79	39.56 (22.44 to 58.38)	-0.28 (-0.76 to 0.21)
22 (lvPPA)				54	0.54	72.97 (54.38 to 87.66)	0.64 (0.11 to 1.16)	38	0.26	13.08 (3.48 to 28.85)	-1.20 (-1.82 to -0.56)
19 (tAD)		55.78 (4.31)	9	56	0.96	51.88 (27.30 to 75.93)	0.05 (-0.60 to 0.70)	40	**0.008**	0.42 (0.00 to 3.78)	-3.66 (-5.53 to -1.78)
24 (tAD)				61	0.28	85.81 (62.30 to 98.05)	1.21 (0.31 to 2.07)	55	0.86	43.41 (20.22 to 68.69)	-0.18 (-0.83 to 0.48)
27 (tAD)				58	0.64	68.09 (42.13 to 88.48)	0.52 (-0.20 to 1.20)	62	0.21	89.59 (68.00 to 99.12)	1.44 (0.47 to 2.37)
5 (lvPPA+)	Synonym judgement task (48)	39.12 (8.41)	17	43	0.66	67.01 (48.13 to 83.03)	0.46 (-0.05 to 0.96)	29	0.26	12.97 (3.43 to 28.81)	-1.20 (-1.82 to -0.56)
18 (lvPPA)				9	**0.003**	0.15 (0.001 to 1.20)	-0.35 (-4.89 to -2.26)	4	**0.001**	0.05 (0.00 to 0.39)	-4.18 (-5.68 to -2.66)
22 (lvPPA)				39	0.99	49.46 (31.23 to 67.78)	-0.01 (-0.49 to 0.46)	35	0.64	32.03 (16.20 to 50.86)	-0.49 (-0.99 to 0.02)
19 (tAD)		44.56 (2.79)	9	46	0.64	68.13 (42.17 to 88.50)	0.52 (-0.20 to 1.20)	40	0.16	7.99 (0.42 to 27.66)	-1.63 (-2.64 to -0.59)
24 (tAD)				43	0.61	30.52 (10.57 to 56.47)	-0.56 (-1.15 to 0.16)	39	0.10	4.77 (0.09 to 20.74)	-1.99 (-3.13 to -0.82)
27 (tAD)				48	0.28	86.21 (62.87 to 98.19)	1.23 (0.33 to 2.09)	48	0.28	86.21 (62.87 to 98.19)	1.23 (0.33 to 2.09)
18 (lvPPA)	ACE-R irregular word reading (5)	3.25 (1.64)	16	1	0.20	10.16 (2.02 to 25.16)	-1.37 (-2.05 to -0.67)	1	0.20	10.16 (2.02 to 25.16)	-1.37 (-2.05 to -0.67)
22 (lvPPA)				5	0.32	84.15 (66.86 to 95.28)	1.07 (0.44 to 1.67)	1	0.20	10.16 (2.02 to 25.16)	-1.37 (-2.05 to -0.67)
19 (tAD)		4.44 (1.57)	9	1	0.07	3.57 (0.03 to 17.47)	-2.19 (-3.41 to -0.94)	1	0.07	3.57 (0.03 to 17.47)	-2.19 (-3.41 to -0.94)
24 (tAD)				5	0.74	62.81 (37.09 to 84.66)	0.36 (-0.33 to 1.02)	5	0.74	62.81 (37.09 to 84.66)	0.36 (-0.33 to 1.02)
27 (tAD)				5	0.74	62.81 (37.09 to 84.66)	0.36 (-0.33 to 1.02)	5	0.74	62.81 (37.09 to 84.66)	0.36 (-0.33 to 1.02)
**Neuroimaging analysis**											
18 (lvPPA)	pSTG-IFG FC z(r)	0.36 (0.25)	15	0.40	0.88	56.05 (36.24 to 74.76)	0.16 (-0.35 to 0.67)	0.44	0.76	61.94 (41.88 to 79.79)	0.32 (-0.21 to 0.83)
22 (lvPPA)				0.40	0.88	56.05 (36.24 to 74.76)	0.16 (-0.35 to 0.67)	0.62	0.33	83.45 (65.28 to 95.15)	1.04 (0.39 to 1.66)
24 (tAD)		0.27 (0.25)	6	0.69	0.18	90.96 (64.13 to 99.83)	1.78 (0.36 to 2.94)	0.38	0.70	64.98 (33.58 to 89.69)	0.44 (-0.42 to 1.26)
27 (tAD)				0.14	0.65	32.54 (8.74 to 64.08)	-0.52 (-1.36 to 0.36)	0.18	0.75	37.63 (12.05 to 68.60)	-0.36 (-1.17 to 0.49)
18 (lvPPA)	pSTG-ATL FC z(r)	0.20 (0.12)	15	0.14	0.64	31.80 (15.17 to 51.87)	-0.50 (-1.03 to 0.05)	0.42	0.10	95.12 (83.61 to 99.61)	1.83 (0.98 to 2.66)
22 (lvPPA)				0.19	0.94	46.85 (27.82 to 66.47)	-0.08 (-0.59 to 0.43)	0.36	0.22	89.12 (73.12 to 97.84)	1.33 (0.62 to 2.02)
24 (tAD)		0.12 (0.30)	6	0.38	0.46	77.06 (45.24 to 96.34)	0.87 (-0.12 to 1.79)	0.26	0.68	65.82 (34.33 to 90.23)	0.47 (-0.40 to 1.30)
27 (tAD)				0.44	0.37	81.56 (50.34 to 98.04)	1.07 (0.008 to 2.06)	0.31	0.58	70.85 (38.96 to 93.25)	0.63 (-0.28 to 1.50)
18 (lvPPA)	pSTG-dPM FC z(r)	0.43 (0.20)	15	0.37	0.78	38.79 (20.84 to 58.83)	-0.30 (-0.81 to 0.22)	0.48	0.81	59.39 (39.42 to 77.65)	0.25 (-0.27 to 0.76)
22 (lvPPA)				0.59	0.45	77.43 (58.05 to 91.51)	0.80 (0.20 to 1.37)	0.36	0.74	36.99 (19.34 to 57.07)	-0.35 (-0.87 to 0.18)
24 (tAD)		0.36 (0.17)	6	0.53	0.37	81.47 (50.24 to 98.91)	1.06 (0.006 to 2.06)	0.41	0.78	60.81 (29.99 to 86.89)	0.31 (-0.53 to 1.12)
27 (tAD)				0.14	0.26	12.95 (0.63 to 42.25)	-1.38 (-2.50 to -0.20)	0.33	0.87	43.46 (16.20 to 73.55)	-0.19 (-0.99 to 0.63)
18 (lvPPA)	IFG-ATL FC z(r)	0.36 (0.20)	15	0.34	0.92	46.22 (27.26 to 65.89)	-0.10 (-0.61 to 0.41)	0.47	0.60	69.87 (49.84 to 86.11)	0.55 (-0.004 to 1.09)
22 (lvPPA)				0.80	**0.05**	97.43 (89.15 to 99.91)	2.20 (1.24 to 3.14)	0.57	0.33	83.67 (65.60 to 95.27)	1.05 (0.40 to 1.67)
24 (tAD)		0.36 (0.09)	6	0.40	0.70	65.12 (33.70 to 89.78)	0.44 (-0.42 to 1.27)	0.43	0.50	74.81 (42.87 to 95.33)	0.78 (-0.18 to 1.68)
27 (tAD)				0.18	0.12	6.17 (0.03 to 29.67)	-2.00 (-3.42 to -0.53)	0.19	0.14	7.04 (0.06 to 31.69)	-1.89 (-3.25 to -0.48)
18 (lvPPA)	IFG-dPM FC z(r)	0.40 (0.21)	15	0.17	0.31	15.35 (4.20 to 33.12)	-1.10 (-1.73 to -0.44)	0.49	0.68	65.78 (45.66 to 92.92)	0.43 (-0.11 to 0.95)
22 (lvPPA)				0.58	0.42	78.97 (59.85 to 92.52)	0.86 (0.25 to 1.44)	0.63	0.31	84.65 (66.84 to 95.79)	1.10 (0.44 to 1.73)
24 (tAD)		0.22 (0.20)	6	0.39	0.47	76.65 (44.80 to 96.16)	0.85 (-0.13 to 1.77)	0.29	0.76	62.06 (31.03 to 87.74)	0.35 (-0.50 to 1.16)
27 (tAD)				0.34	0.60	69.88 (38.04 to 92.70)	0.60 (-0.30 to 1.45)	0.37	0.52	74.08 (42.13 to 94.96)	0.75 (-0.20 to 1.64)

18 (lvPPA)	ATL-dPM FC z(r)	0.09 (0.15)	15	0.17	0.61	69.32 (49.27 to 85.69)	0.53 (-0.02 to 1.07)	0.24	0.35	82.53 (64.13 to 94.65)	1.00 (0.36 to 1.61)
22 (lvPPA)				0.35	0.12	94.22 (81.80 to 99.43)	1.73 (0.91 to 2.53)	0.20	0.49	75.53 (55.92 to 90.24)	0.73 (0.15 to 1.30)
24 (tAD)		0.06 (0.25)	6	0.31	0.40	80.15 (48.68 to 97.56)	1.00 (-0.03 to 1.97)	0.35	0.33	83.40 (52.61 to 98.58)	1.16 (0.07 to 1.19)
27 (tAD)				0.09	0.92	54.22 (24.54 to 82.09)	0.12 (-0.69 to 0.92)	0.31	0.40	80.15 (48.68 to 97.56)	1.00 (-0.03 to 1.97)

Significant two-tailed probabilities are indicated in bold font. While the overall group sample sizes were nine and 18 for tAD and lvPPA/lvPPA+, respectively, we included a column for "Group sample size" as a few patients did not complete some tests, affecting the sample size for those tests. ACE-R, Addenbrooke's Cognitive Examination – Revised; ATL, anterior temporal lobe; BNT, Boston Naming Test; CSB, Cambridge Semantic Battery; dPM, dorsal premotor; FC, functional connectivity; IFG, inferior frontal gyrus; lvPPA, logopenic variant primary progressive aphasia; PALPA, Psycholinguistic Assessment of Language Processing in Aphasia; pSTG, posterior superior temporal gyrus; SD, standard deviation; tAD, typical Alzheimer's disease.

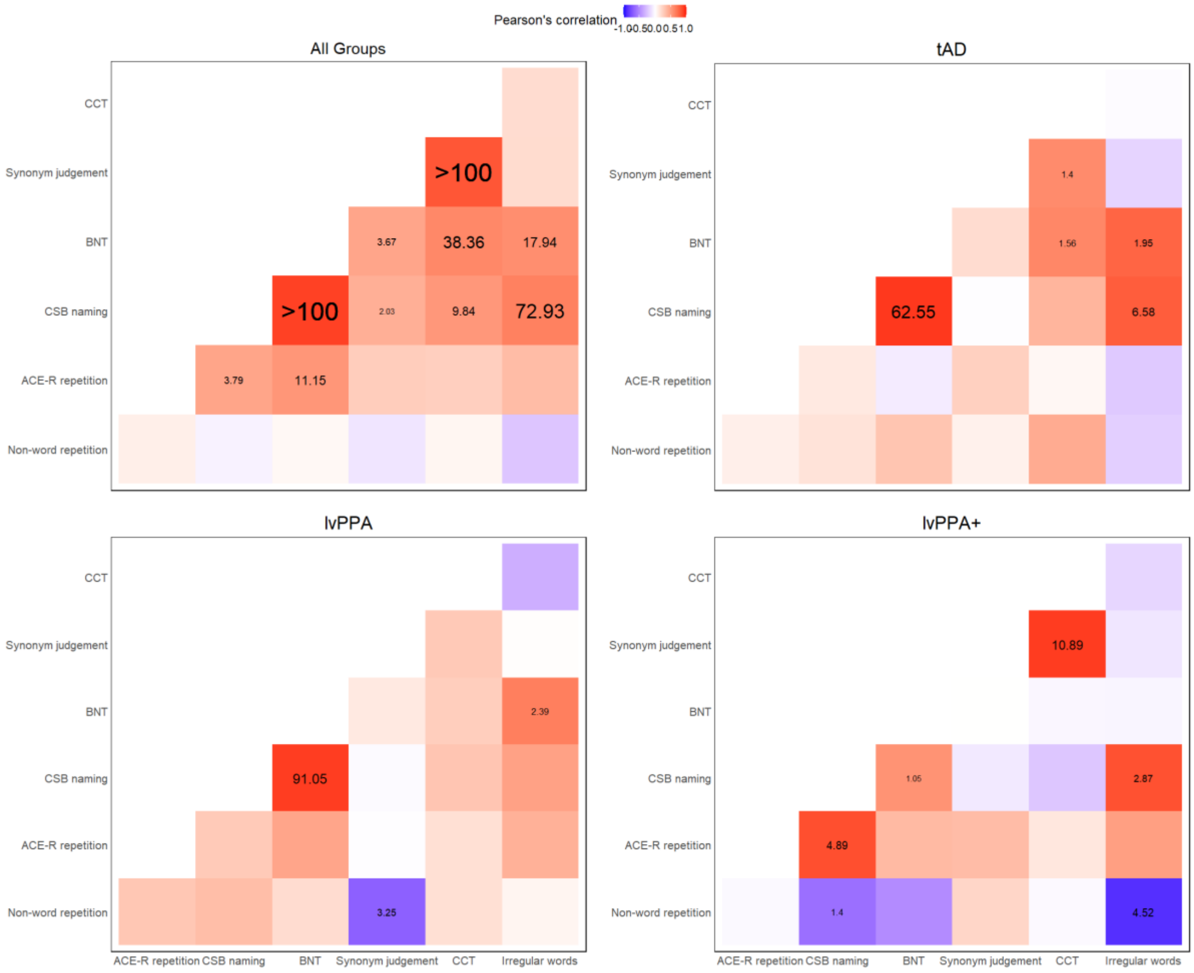

Supplementary Figure 1 Associations between PALPA non-word and ACE-R multisyllabic word/phrase repetition and semantic assessments. Pearson's correlation values are represented in the colour legend, where red and purple colours represent positive and negative correlations, respectively. The associated Bayes factors (BFs) are shown in black text, where the larger versus smaller fonts denote the degree of evidence (e.g., extreme for BF >100 versus anecdotal for 1 < BF < 3). In the individual patient groups, the only other moderate evidence was found between non-word repetition and synonym judgement task in the lvPPA group (r = -0.69, P = 0.03, BF = 3.25), as well as between non-word repetition and ACE-R irregular word reading (r = -0.89, P = 0.02, BF = 4.52) and between ACE-R multisyllabic word/phrase repetition and CBS naming (r = 0.84, P = 0.04, BF = 4.89) in the lvPPA+ group.

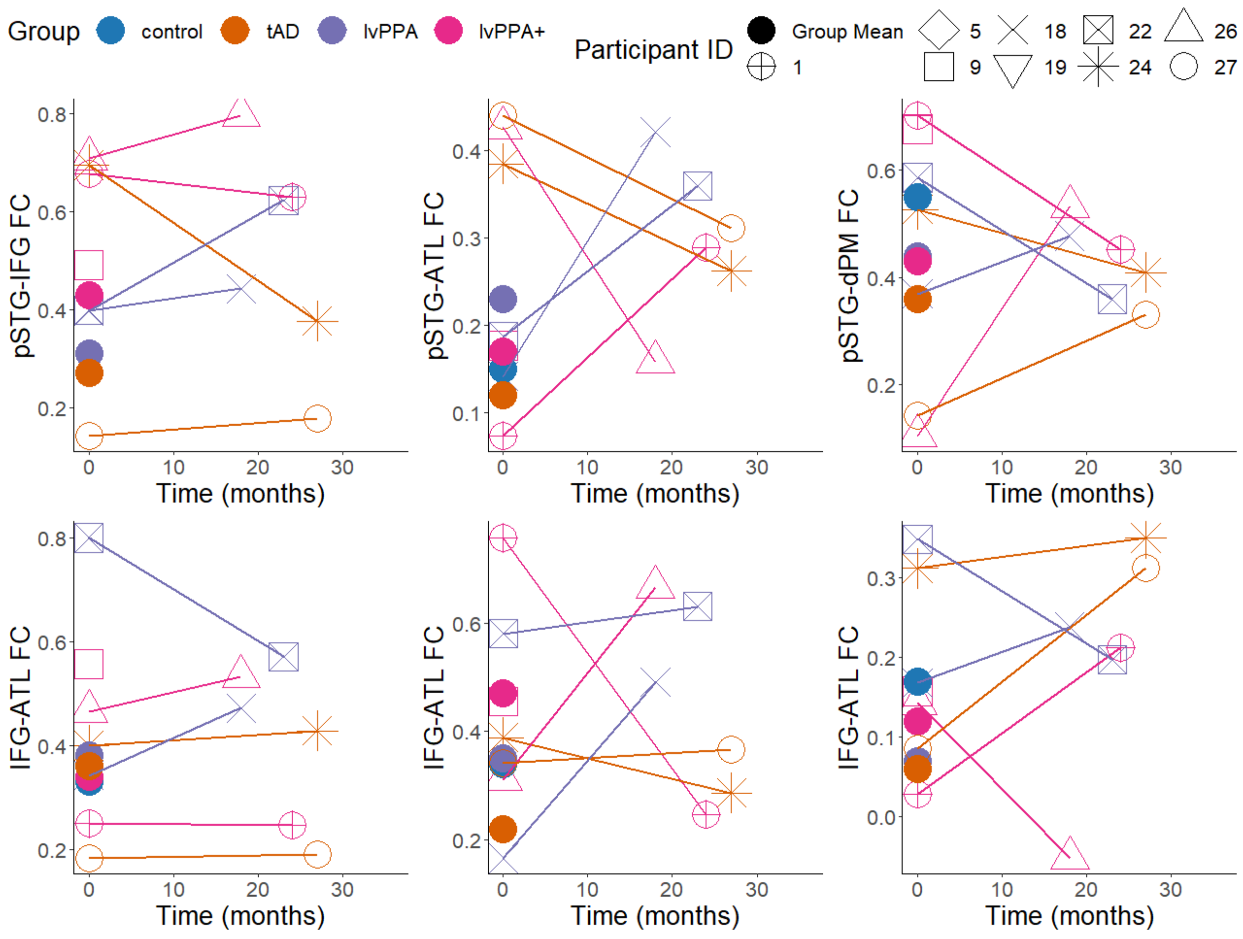

Supplementary Figure 2 Functional connectivity z-values between all region-of-interests (ROIs) over time in six patient participants. The x-axis represents the time from to initial to follow-up assessment in months. The filled colour dots represent the group average functional connectivity z-values for each ROI pair. The connected lines indicate the scores of each participant who completed the same test at two time-points. Note: The lines merely connect the same participants across two time-points and do not suggest a linear decline over time. ATL, anterior temporal lobe; dPM, dorsal premotor region; FC, functional connectivity; IFG, inferior frontal gyrus, lvPPA, logopenic variant of primary progressive aphasia; pSTG, posterior superior temporal gyrus; tAD, typical Alzheimer's disease.
